# The association between TyG index and hypertension in middle-aged and elderly Chinese patients: Data from CHALRS

**DOI:** 10.1371/journal.pone.0329234

**Published:** 2025-07-28

**Authors:** Jun Wang, Chao Tang, Zhijie Xie

**Affiliations:** 1 Department of Neurosurgery, Master of Medicine, The First People’s Hospital of Linping District, Hangzhou, Zhejiang Province, China; 2 Department of Neurosurgery, Bachelor of Medicine, The First People’s Hospital of Linping District, Hangzhou, Zhejiang Province, China; Tehran University of Medical Sciences, IRAN, ISLAMIC REPUBLIC OF

## Abstract

**Background:**

The TyG index is thought to be a trustworthy substitute indicator of insulin resistance. Increasing research evidence shows the correlation between TyG and various cardiovascular and cerebrovascular diseases and adverse prognosis. However, the effect of diabetes on the connection between TyG and hypertension has not been extensively studied. In order to identify high-risk individuals, our research aimed to investigate the potential relationship between the TyG index and the risk of hypertension in middle-aged and elderly Chinese individuals.

**Methods:**

This study analyzed and collected information of the middle-aged and elderly population from the 2015 China Health and Retirement Longitudinal Study (CHARLS) database, and three groups were created based on the tertiles of TyG. First, the clinical characteristics of patients in different groups were analyzed univariately, and logistic regression analysis and RCS model were utilized to further clarify the relationship between the TyG index and hypertension. Finally, subgroup analysis was performed to distinguish the effects of different baseline characteristics on the connection between TyG and hypertension.

**Results:**

9695 patients in all were enrolled, including 4548 males (46.9%) and 5147 females (53.1%). The incidence of hypertension in all patients was 33.7%. As displayed in Table 1, as the TyG index rises, the incidence of Diabetes, Heart disease, and Stroke in patients increased accordingly. Similarly, in terms of laboratory indicators, White blood cell, Platelets, Triglycerides, Uricacid, and Hbg increased with the rises of TyG; while the incidence of lung diseases, BUN, and HDL levels showed a downward trend. The RCS model showed that there was a statistically significant nonlinear association between TyG and hypertension (p value <0.001, nonlinear p = 0.008); Subgroup analysis showed that different baseline characteristics may influence the association between TyG and hypertension risk.

**Conclusion:**

Our study’s findings demonstrate a substantial correlation between TyG index and hypertension, showing a positive correlation in both adjusted and unadjusted logistic regression models, which may help identify individuals at risk for hypertension and have great potential through early improvement of blood pressure management. It has great potential to reduce the occurrence related to cardiovascular and cerebrovascular disorders.

## 1. Introduction

Hypertension is a prevalent risk factor for cardiovascular and cerebrovascular disorders [1]. The number of people suffering from hypertension has doubled during the previous few decades [2]. Comprehensive survey results show that there are no less than 200 million people with hypertension in China, and more than 1.3 billion people in the world suffer from hypertension [3,4]. As the understanding of hypertension continues to deepen, the treatment of hypertension has also been continuously improved, but the blood pressure control of the population is not ideal [5,6]. Many people with high blood pressure do not show obvious symptoms, but brain hemorrhage and ruptured aneurysms caused by high blood pressure can be fatal. Other complications like coronary artery disease can also be very dangerous and fatal [7]. Therefore, it is crucial to further explore the risk factors of hypertension and take preventive measures.

Insulin resistance (IR) refers to the reduced sensitivity and reactivity to the physiological actions of insulin, which is often closely associated with metabolic syndrome. It is accompanied by abnormal glucose and lipid metabolism, thus promoting the incidence of various cardiovascular and cerebrovascular diseases, and is strongly linked to a poor prognosis [8–12]. Despite being the gold standard for diagnosing insulin resistance, the glucose clamp technique’s extensive use in clinical practice is limited by its complex and costly procedure [13].

The TyG index is increasingly acknowledged as a trustworthy substitute marker for IR, due to its convenience and affordability [14]. There is a strong association between the rise in TyG index and the severity of glucose and lipid metabolism abnormalities, and also with the growing occurrence of metabolic syndrome in patients. Convincing empirical results from numerous investigations have solidified the correlation between TyG and the incidence and prognosis of cerebrovascular and cardiovascular disorders [15,16]. Another prospective cohort study has also provided more clarification that TyG index serves as a potential indicator of cardiovascular disease risk within populations without diabetes [17]. Nevertheless, limited research has specifically examined the influence of diabetes on the association between TyG and hypertension. Thus, our study sought to use data from CHARLS to evaluate, in middle-aged and elderly Chinese people, the relationship between TyG levels and hypertension, which would be beneficial for early identification and tracking of hypertension and have important implications for early management of hypertension.

## 2. Methods

### 2.1. Study population

This retrospective analysis utilized the public dataset of the China Health and Retirement Longitudinal Study (CHARLS), which was conducted in 2011 and covered 150 county-level units, 450 village-level units, and 17,000 people in approximately 10,000 households. It fully represents the high-quality information of families and individuals of middle-aged and elderly people aged 45 and above in China, and contains detailed information on family demographics, household composition, and health status.

**Fig 1:**
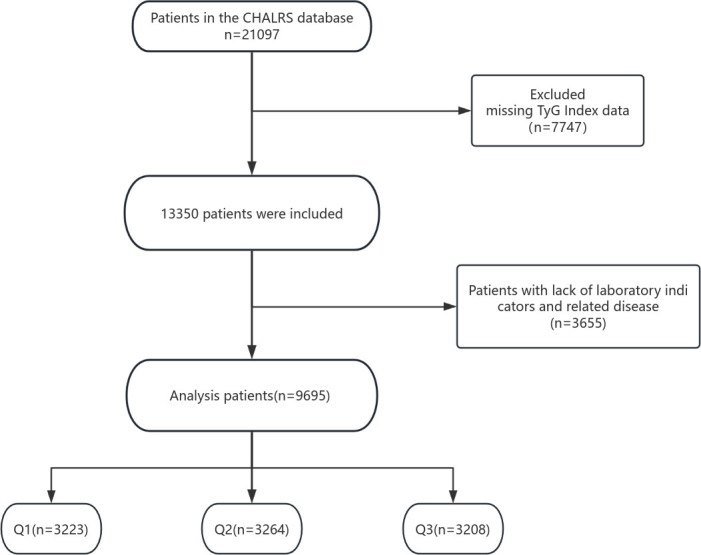
Flow chart.

The analysis used data from the 2015 CHARLS survey. In total, 9695 subjects aged 45 years or older were enrolled, including 4548 males and 5147 females. Patients with missing clinical data were excluded, and the TyG index was computed utilising relevant laboratory indicators. The patients were categorized and grouped based on the tertiles of TyG. See Fig 1 for details.

This research involves the CHARLS public database. The local Peking University Biomedical Ethics Review Committee has agreed to collect CHARLS data (IRB00001052–11015), and all participants have signed the informed consent form. The research was conducted in accordance with relevant guidelines/regulations, and the method was in line with the ethical guidelines of Helsinki Declaration.

### 2.2. Definition

Definition of hypertension [18]:(1)Mean diastolic blood pressure ≥90 mmHg or a mean systolic blood pressure ≥140 mmHg; (2) patient’s self-stated hypertension; (3) taking antihypertensive medications as prescribed. These criteria adhere to the standards established by the International Society of Hypertension.

Based on fasting blood glucose and triglyceride levels, the Tyg index is determined by employing the subsequent formula:


TyG\Index=ln[FBG*TG/2]

TG = fasting triglycerides in mg/dL; FBG = fasting blood glucose in mg/dL; ln = natural logarithm

### 2.3. Statistical analysis

All analyses were performed utilising the statistical software SPSS26. For data collection, variables with missing data rates greater than 10% were eliminated, variables with missing data rates between 5% and 10% were interpolated using mean interpolation. For variables with a missing rate below 5%, the missing variable is deleted. Initially,the triple digits of the TYG index were calculated based on the 33% and 66th percentile distribution in the study population. Patients were divided into three groups: Q1 (≦8.38), Q2 (8.38 ~ 8.92) and Q3 (>8.92). Kolmogorov–Smirnov test was utilized to test the normal distribution of continuous variables, and the results showed that the TyG index showed a skewed distribution. And the Kruskal-Wallis test was utilized for non-normally distributed continuous data using the median (IQR),the chi-square test was employed to analyze categorical variables, which were presented as n (%). Unadjusted and adjusted logistic regression models were employed to evaluate the link between TyG and hypertension. In smaller modified models, we controlled simply for BMI, age, and diabetes. In the fully adjusted model, all covariates included chronic diseases (Diabetes, Stroke, Heart disease, Lung disease, Arthritis), Age, BMI, WBC, MCV, BUN, Creatinine, Triglycerides, HDL, Uric acid, Hbg. A multicollinearity test was performed on the variables included in the model, and the variance inflation factor VIF was calculated. When VIF < 5, it was considered to have no significant collinearity characteristics. The OR value and 95%CI of the logistic regression model were computed, P < 0.05 was considered statistically significant.In addition, we performed subgroup analysis to distinguish the clinical applicability of TyG under different baseline features.

## 3. Result

### 3.1. Baseline characteristics

This investigation included 9695 patients in total, who were categorized based on the TyG Index tertiles. Among them, there were 4548 males (46.9%) and 5147 females (53.1%). The incidence of hypertension was 33.7% in the enrolled patients. According to Table 1, as TyG rises, the incidence of Diabetes(p < 0.001), Heart disease(p < 0.001), Stroke(p = 0.002) in patients increased accordingly. Similarly, in terms of blood pressure and laboratory indicators, WBC(p < 0.001), Plt(p < 0.001), Triglycerides(p < 0.001), Uric acid(p < 0.001), and Hbg(p < 0.001) increased with the rise of TyG; While the incidence of Lung diseases(p = 0.002), BUN(p < 0.001), and HDL(p < 0.001) levels showed a downward trend.

**Table 1:** Individual characteristics.

Characteristic Triglyceride-glucose index					p
	Totall	Q1(≦8.38)	Q2(8.38 ~ 8.92)	Q3(>8.92)	
	N = 9695	N = 3223	N = 3264	N = 3208	
Age (years)	61.0(53.0 ~ 67.0)	61.0(53.0 ~ 68.0)	61.0(54.0 ~ 68.0)	60.0(53.0 ~ 66.0)	<0.001
Sex					<0.001
Male	4548(46.9%)	1725(53.5%)	1450(44.8%)	1373(42.4)	
Female	5147(53.1%)	1498(46.5%)	1786(55.2%)	1863(57.6%)	
Diabetes	964(9.9%)	130(4.0%)	233(7.2%)	601(18.6%)	<0.001
Hypertension	3266(33.7%)	797(24.7%)	1063(32.8%)	1406(43.4%)	<0.001
Cancer	161(1.7%)	43(1.3%)	55(1.7%)	63(1.9%)	0.153
Lung disease	1329(13.7%)	490(15.2%)	446(13.8%)	393(12.1%)	0.002
Heart disease	1739(17.9%)	487(15.1%)	580(17.9%)	672(20.8%)	<0.001
Stroke	321(3.3%)	86(2.7%)	100(3.1%)	135(4.2%)	0.002
Arthritis	4172(43.0%)	1392(43.2)	1397(43.2)	1383(43.2)	0.918
Liver Disease	625(6.4%)	217(6.7%)	203(6.3)	205(6.3%)	0.717
Kidney disease	935(9.6%)	332(10.3%)	299(9.2%)	304(9.4%)	0.296
Stomach disease	2997(30.9%)	1033(32.1%)	1008(31.1%)	956(29.5%)	0.087
Asthma	561(5.8%)	185(5.7%)	206(6.4%)	170(5.3%)	0.158
Systolic blood pressure (mmHg)	125.5 (113.0 ~ 140.0)	122.0 (109.5 ~ 150.5)	125.0 (113.0 ~ 139.5)	129.0 (117.0 ~ 143.0)	<0.001
Diastolic blood pressure (mmHg)	74.0 (67.0 ~ 82.5)	72.0 (65.0 ~ 80.5)	74.0 (67.0 ~ 82.0)	76.0 (69.0 ~ 84.0)	<0.001
Body Mass Index (kg/m^2^)	23.67 (21.33 ~ 26.28)	22.08 (20.02 ~ 24.44)	23.72 (21.45 ~ 25.99)	25.25 (22.98 ~ 27.69)	<0.001
White Blood Cell(10^9/L)	5.70 (4.77 ~ 6.90)	5.40 (4.50 ~ 6.57)	5.70 (4.80 ~ 6.80)	6.05 (5.07 ~ 7.23)	<0.001
Mean Corpuscular Volume (fl)	92.0 (88.1 ~ 95.9)	92.7 (88.3 ~ 96.5)	92.0 (88.4 ~ 95.7)	91.7 (87.8 ~ 95.3)	<0.001
Blood Urea Nitrogen (mg/dl)	14.85 (12.32 ~ 18.21)	15.41 (12.61 ~ 18.49)	14.85 (12.32 ~ 17.93)	14.57 (12.32 ~ 17.65)	<0.001
Platelets (10^^9^/L)	201(159 ~ 243)	195(155 ~ 236)	203(159 ~ 247)	205(164 ~ 249)	<0.001
Creatinine (mg/dl)	0.76(0.66 ~ 0.89)	0.77(0.67 ~ 0.89)	0.77(0.67 ~ 0.90)	0.75(0.64 ~ 0.90)	<0.001
Triglycerides (mg/dl)	114.16 (83.19 ~ 169.03)	74.34 (61.95 ~ 84.96)	115.93 (100.89 ~ 134.51)	204.42 (166.37 ~ 273.45)	<0.001
High-density lipoprotein (mg/dl)	49.81 (43.24 ~ 57.53)	54.83 (47.49 ~ 62.93)	50.58 (44.02 ~ 57.53)	45.56 (40.15 ~ 51.74)	<0.001
Low-density lipoprotein (mg/dl)	101.16 (83.01 ~ 119.31)	94.98 (79.15 ~ 112.36)	106.18 (88.42 ~ 124.71)	101.16 (82.63 ~ 121.62)	<0.001
Uric acid (mg/dl)	4.8(3.9 ~ 5.7)	4.5(3.7 ~ 5.4)	4.8(4.0 ~ 5.7)	5.1(4.3 ~ 6.1)	<0.001
Hemoglobin (g/dl)	13.6(12.5 ~ 14.8)	13.4(12.3 ~ 14.6)	13.6(12.6 ~ 14.8)	13.8(12.7 ~ 15.0)	<0.001
TyG Index	8.62(8.25 ~ 9.09)	8.11(7.93 ~ 8.25)	8.62(8.50 ~ 8.76)	9.32(9.09 ~ 9.69)	<0.001

Skewed data were displayed as median (IQR), whereas categorical data were given as n(%).

TyG, the triglycerides and glucose index.

### 3.2. Associations between TyG and hypertension

Univariate regression analysis was performed on the above variables, and variables (p < 0.05) were included in the multiple logistic regression model. Table 2 displays the multivariate regression analysis’s correlation between TyG and hypertension. The results show the OR values when TyG is used as a categorical and continuous variable. In model 2, covariate included age, BMI, diabetes. When considering as a categorical data, compared to Q1 group, the OR value of hypertension risk in the Q2 group was 1.44 (1.29 ~ 1.62), and the OR value of hypertension risk in the Q3 group was 2.08 (1.86 ~ 2.32). When considering as a continuous data, the risk of hypertension increased by 1.56 (1.46 ~ 1.68) times for each unit rise in the TyG Index (P < 0.001). In model 3, all adjustment variables were included. Compared with group Q1, the OR of hypertension in group Q2 was 1.32(1.17 ~ 1.49), and the OR of hypertension in group Q3 was 1.72(1.45 ~ 2.02). As a continuous variable, the risk of hypertension increased by 1.57(1.34 ~ 1.84) times for each unit rise in the TyG Index (P < 0.001). In general, higher TyG index is positively correlated with the occurrence of hypertension.

**Table 2:** Connection between TyG and risk of hypertension.

Models	TyG Index			TyG Index (Constant)	P
	Q1(≦8.38)	Q2(8.38 ~ 8.92)	Q3(>8.92)		
Model 1 OR(95%CI)	Ref	1.49(1.34 ~ 1.66)	2.34(2.10 ~ 2.60)	1.71(1.60 ~ 1.83)	<0.001
Model 2 OR(95%CI)	Ref	1.44(1.29 ~ 1.62)	2.08(1.86 ~ 2.32)	1.56(1.46 ~ 1.68)	<0.001
Model 3 OR(95%CI)	Ref	1.33(1.18 ~ 1.50)	1.74(1.54 ~ 1.97)	1.38(1.28 ~ 1.50)	<0.001

Model 1: unadjusted model.

Model 2: adjusted for Age, BMI, Diabetes.

Model 3: adjusted for Age, BMI, Diabetes, Stroke, Heart disease, Lung disease, Arthritis, WBC, MCV, BUN, Creatinine, Triglycerides, HDL, Uric acid, Hbg.

**Fig 2:**
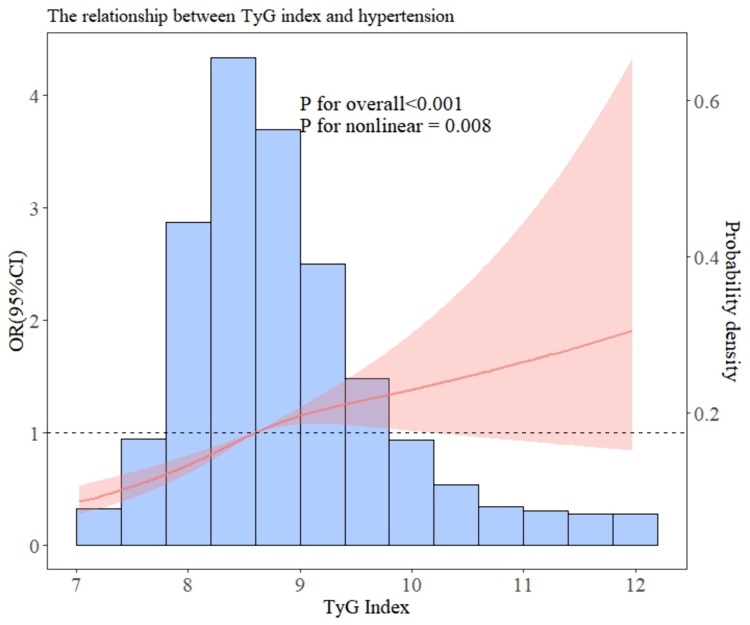
RCS curve is the odds ratio of TyG index. All covariates included in the model include: Age, Diabetes, Stroke, Heart disease, Lung disease, Arthritis, BMI, WBC, MCV, BUN, Creatinine, Triglycerides, HDL, Uric acid, Hbg. OR: odds ratio; CI, confidence interval; RCS, restricted cubic spline.

After including all covariates, including: Age, Diabetes,Stroke, Heart disease, Lung disease, Arthritis, BMI, WBC, MCV, BUN, Creatinine, Triglycerides, HDL, Uric acid, Hbg. The RCS curve analysis in Fig 2 demonstrated a nonlinear association between TyG and hypertension (p value < 0.05, nonlinear p < 0.05).

### 3.3. Subgroup analysis

**Fig 3:**
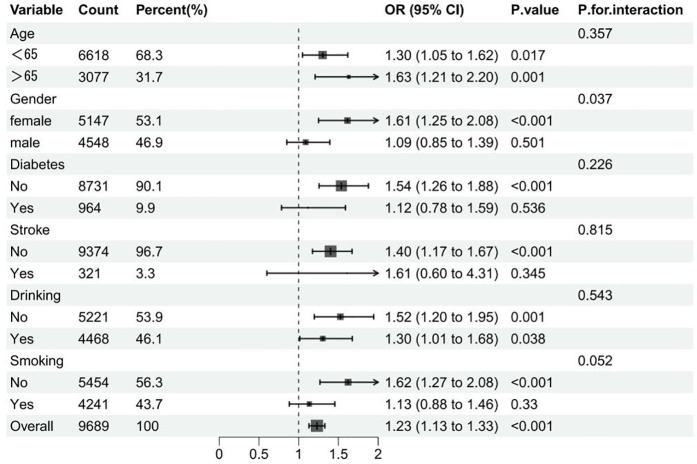
Subgroup analysis of correlation between TYG index and hypertension.

**Fig 4:**
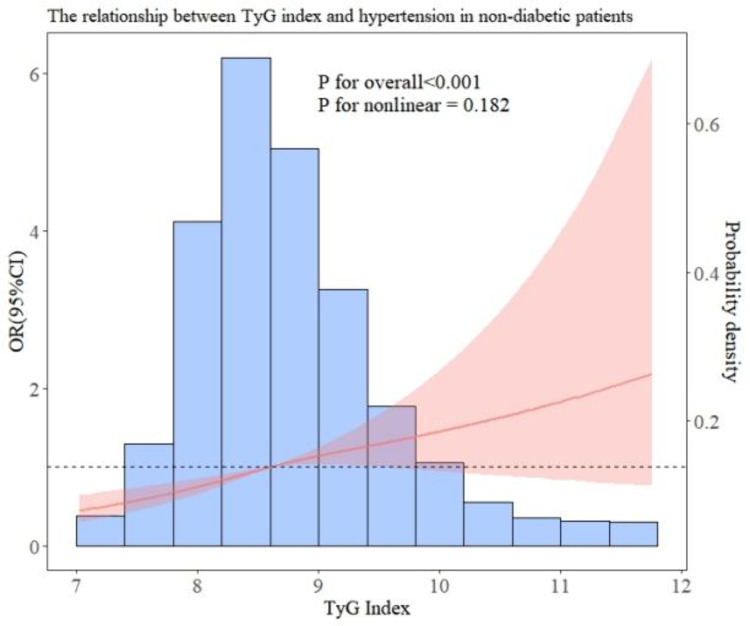
Represents the hypertension risk curve for non-diabetic patients.

**Fig 5:**
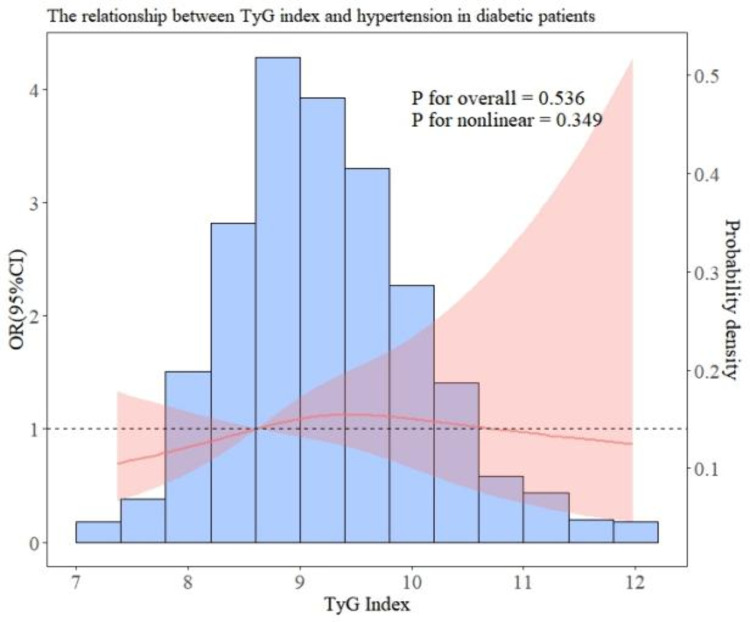
Represents the hypertension risk curve for diabetic patients.

**Fig 6:**
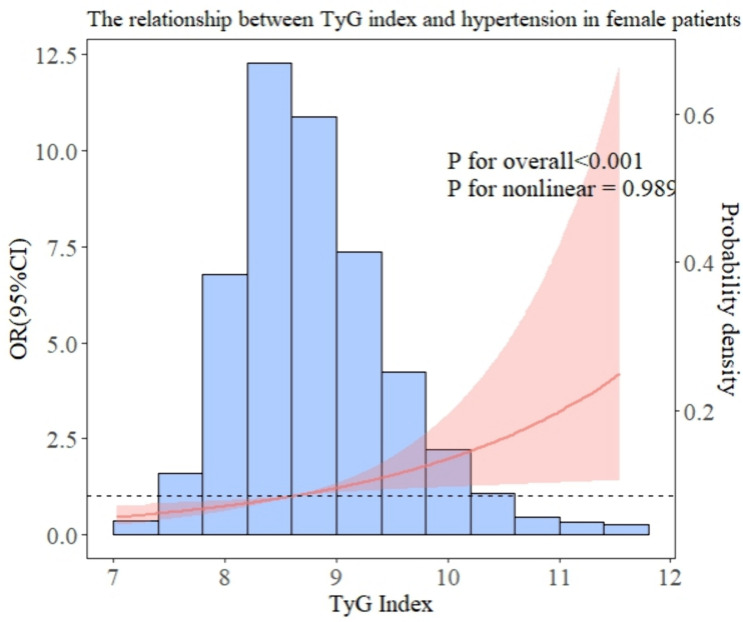
Represents the hypertension risk curve for female patients.

**Fig 7:**
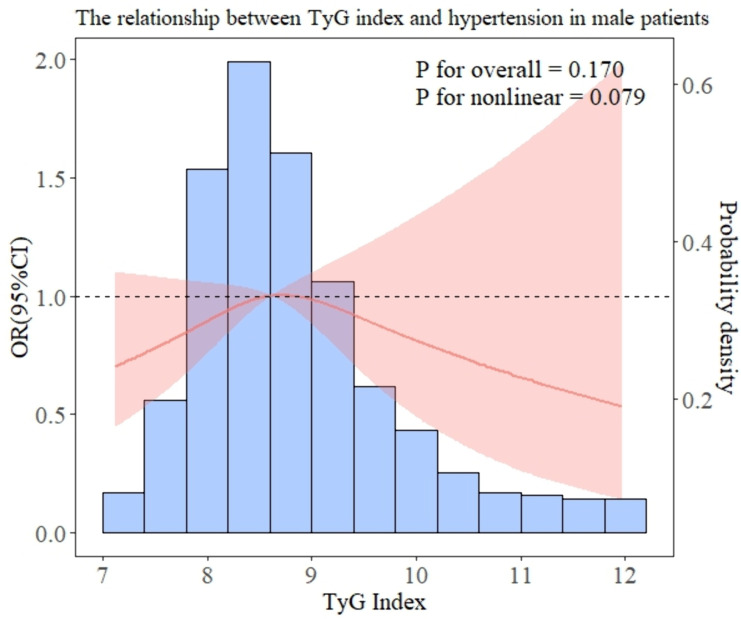
Represents the hypertension risk curve for male patients.

In addition, we performed subgroup analyses to examine the association between TyG and hypertension in patients with different baseline characteristics. After all covariables were included in model 3, Fig 3 showed the OR value of interaction between TyG index and hypertension and P for interaction of each subgroup. As can be seen in Fig 3, there is a significant interaction in gender (interaction P = 0.037), which may affect the relationship between TyG index and hypertension risk. The results of the forest map show that the OR values of all subgroups are greater than 1, indicating that the risk of hypertension increases with the increase of TyG index. Overall, there was no significant interaction between most groups. In addition, the RCS relationship between TyG index and hypertension under several common different baseline characteristics is drawn. As shown in Figs 4 and 5, there is no significant correlation between the risk of hypertension and TyG in diabetic patients, but in non-diabetic patients, the TyG index is linearly positively correlated with hypertension. In Figs 6 and 7, there is a positive linear correlation between the risk of hypertension and TyG in female patients, but there is no significant correlation between the TyG index and hypertension in male patients.

## 4. Discussion

In this cross-sectional study based on middle-aged and elderly Chinese population. We examined the correlation between hypertension and TyG, and our findings demonstrated a significant association between higher TyG and the risk of hypertension. Furthermore, the prevalence of diabetes, heart disease, and stroke had a positive correlation with TyG. The strong correlation between TyG and hypertension persisted even after accounting for traditional risk variables. According to these results, the TyG index is independently correlated with hypertension and could be used to monitor and identify those who are at risk for the condition. Furthermore, the RCS findings revealed a nonlinearly positive correlation between the risk of hypertension and the alteration in TyG index.

TyG index is an invaluable surrogate indicator of IR that is commonly employed and is determined by fasting triglyceride and blood glucose levels. Increasing research evidence has proven that higher TyG is significantly correlated to diabetes, hypertension, chronic kidney disease, cardiovascular disease, coronary artery calcification, etc [14,19–22]. In our investigation, we also found similar outcomes, which could be connected to the following mechanisms.

IR is a condition in which the body’s capacity to absorb and metabolize glucose and its sensitivity to insulin are diminished. Due to insulin resistance, the increase of free fatty acids in blood, making them more likely to be deposited in other organs such as the liver, cardiovascular, cerebrovascular systems, etc [23]. Many other research have also validated that compensatory hyperinsulinemia is an independent risk factor for atherosclerotic heart disease and hypertension [22,24–26]. Furthermore, insulin resistance is linked to chronic inflammation of the blood vessel wall, oxidative stress, and endothelial dysfunction, which further leads to vascular stiffness and elevated blood pressure [8,27], which may partially explain the increased incidence of cerebrovascular disease and hypertension at the center of this study. Recent research examining the correlation between TyG index and the risk of cardiovascular disease in older adults has discovered a heightened risk of cardiovascular disease, consistent with the findings of this study [28,29]. On this basis, we further performed subgroup analysis and plotted forest maps to explore whether different baseline characteristics would affect the association between TyG and cardiovascular disease.

Numerous epidemiological research have demonstrated that association between high TyG index and hypertension risk and serving as a valid proxy for insulin resistance [30–33]. Interestingly, there was no discernible correlation between TyG index and hypertension within diabetic patients according to the results of our subgroup study. While the precise mechanism remains unclear, the administration of hypoglycemic medications or insulin in diabetic patients can potentially impact the real levels of blood sugar and insulin in the body. Moreover, diabetes can significantly influence the advancement of hypertension and is also a significant risk element [17]. Additional study has indicated that diabetes and hyperlipidemia can potentially impact the accuracy of using the TyG index for evaluating cardiovascular disease [34]. Therefore, TyG may not accurately represent the actual state of glucose and lipid metabolism individuals with diabetes, so the connection between TyG index and hypertension may be masked. Moreover, there seems to be no clear correlation between male TyG index and hypertension risk in different gender subgroups. A study by Lu et al. Gender differences in the relationship between TyG index and atherosclerosis pointed out that differences in fat storage sites in different sexes may affect IR development. Compared with men, women have greater changes in lipid status and metabolic deterioration when increasing the same fat storage level [35]. Additionally, many studies have revealed the correlation between estrogen deficiency and insulin resistance [36]. M R Meyer [37] research pointed out that women are more likely to accelerate obesity after menopause, which may be related to the lack of estrogen after menopause and has been confirmed by many animal experiments [38].

Our research also strengthens this view. The research object is middle-aged and elderly women, and the lack of estrogen is more likely to cause insulin resistance and obesity.

Therefore, it may be necessary to adopt a gender-specific method to identify individuals at risk of hypertension by using TyG index.Actively looking for risk factors related to the development of hypertension and improving blood pressure management have great potential to reduce the occurrence of various disease events such as cardiovascular and cerebrovascular diseases.

Nevertheless, it is important to recognize that this study does have certain constraints. Firstly, our study subjects were middle-aged and elderly Chinese individuals, the findings may lack generalizability to individuals from different age cohorts or diverse ethnic backgrounds. Secondly, as a cross-sectional observational study, we have solely identified a correlation between TyG and hypertension. We cannot exclude the potential of a reverse causal connection between them, which still needs further study. Finally, for different clinical characteristics (including diabetes and gender, etc.), which may affect the correlation between TyG and the risk of cardiovascular diseases such as hypertension, further research is needed to explore the underlying mechanism.

## 5. Conclusion

According to our findings, different baseline characteristics may affect the relationship between TyG index and hypertension risk. Elevated TyG index in women and non-diabetic patients is associated with an increased risk of hypertension, and individualized risk management strategies are still needed for early prevention of hypertension risk.
